# Gap analysis of breast cancer care at tertiary facilities in Armenia

**DOI:** 10.3389/fonc.2026.1907028

**Published:** 2026-09-03

**Authors:** Ani Arzoumanian, Sione Markarian, Anja Johnson, Emma Mastro, Sira Jil-Agopian, Catherine Duggan, Marine Hovhannisyan, Henry Louis, Shant Shekherdimian

**Affiliations:** 1 University of Massachusetts Chan Medical School, Worcester, MA, United States; 2 University of California Los Angeles David Geffen School of Medicine, Los Angeles, CA, United States; 3 V.A. Fanarjian National Center of Oncology, Yerevan, Armenia; 4 Fred Hutchinson Cancer Center, Seattle, WA, United States; 5 Yerevan State Medical University, Department of Hygiene and Ecology, Yerevan, Armenia

**Keywords:** Armenia, breast cancer, breast cancer diagnostic, breast cancer early detection, breast cancer treatment, global health oncology, health system capacity

## Abstract

Breast cancer is the leading cause of cancer mortality among women globally. Armenia has one of the highest breast cancer mortality rates in the world, with an age standardized mortality-to-incidence ratio of 0.41, indicating that two in five breast cancer patients die from the disease. The factors contributing to this disproportionate mortality burden are poorly understood. The present study utilized a validated survey across four high-capacity cancer facilities to produce a national gap analysis of breast cancer care in Armenia. We find that the tertiary facilities surveyed broadly possess the fundamental technological and personnel resources required for modern breast cancer care; however, it is apparent that challenges exist with early detection, timely diagnosis, and successful treatment. Based on these findings, policy considerations could prioritize breast cancer mortality reduction through early detection, establishment of a cancer registry, streamlining cancer care, and reducing the financial burden of care on patients.

## Introduction

Breast cancer has the highest global incidence among cancers and is the leading cause of cancer-related mortality in women (1). Armenia, an Upper-Middle Income Country (UMIC) in the Southern Caucasus, has been reported to have one of the highest breast cancer mortality rates in the world, despite having a lower incidence rate compared to countries with similar economic status, geographic location, and Human Development Index (HDI) (1–3). The age-standardized mortality-to-incidence ratio (MIR) is 0.41 in Armenia, indicating that two in five women with breast cancer will die from the disease (4). This is significantly higher than the MIR of 0.23 for UMIC countries and 0.17 for Very High HDI countries. The causes of the disparity remain unclear, but likely result from an amalgamation of challenges across the care spectrum.

**Figure 1:**
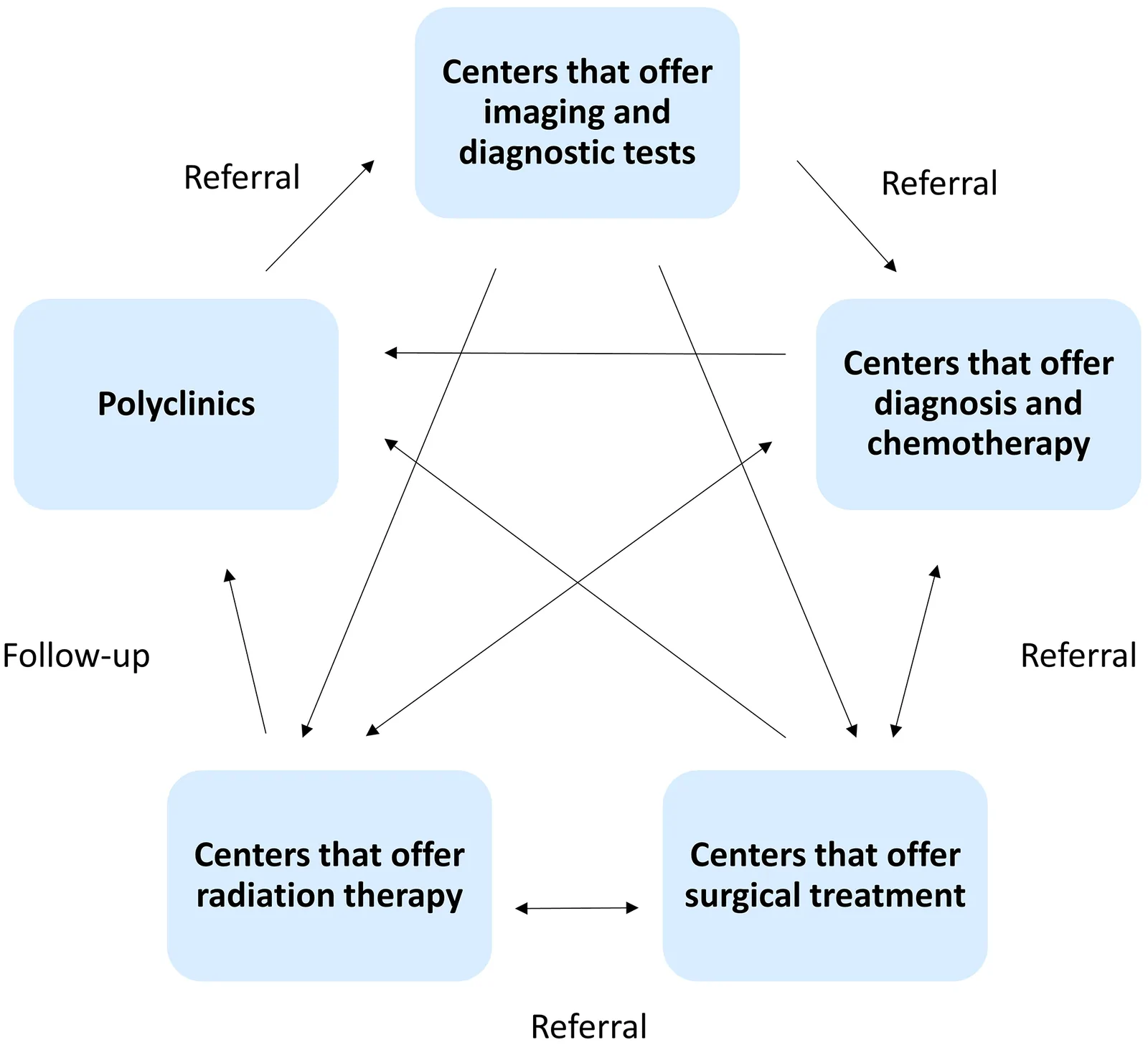
The cancer care pathway is not streamlined in Armenia; thus, breast cancer patients are required to seek diagnostic modalities or components of treatment at separate facilities. There is no standardized order — individual patients will navigate this process in different sequences. Figure 5. Reprinted with permission from "Overview of Cancer Control in Armenia and Policy Implications," by K. Bedirian et al., 2022, licensed under CC BY 4.0, https://www.frontiersin.org/journals/oncology/articles/10.3389/fonc.2021.782581/full.

The cancer diagnosis and treatment process in Armenia is not streamlined, resulting in a complex, multi-institution, and inefficient care pathway which places significant responsibility on the patient to coordinate their own follow-up care (**Figure 1**). Without a national cancer registry, Armenia faces significant challenges in surveillance and policy evaluation (5). While most cancer imaging and treatment modalities are available in Armenia, a lack of standardized protocols leads to discrepancies in diagnostic and treatment methods across care settings (6). Moreover, the financial burden of treatment is often placed on patients, leading to incomplete treatments, and genetic testing is utilized infrequently due to cost (6). Out-of-pocket expenditures in Armenia remain among the highest globally, accounting for as much as 84.8% of current health expenditure in 2019, and although universal health coverage is now being implemented in a phased transition initiated in 2026, the depth of coverage for cancer diagnostics and therapeutics remains undefined (7, 8).

The World Health Organization Global Breast Cancer Initiative (WHO-GBCI) aims to reduce breast cancer mortality globally through three pillars: 1) early detection — at least 60% of invasive cases diagnosed at Stage I/II, 2) timely diagnosis — completion of clinical evaluation, imaging, tissue sampling, and pathology within 60 days, and 3) comprehensive breast cancer management — 80% of patients receiving full multimodal treatment and successful return home (9).

Early detection of disease involves both screening of non-symptomatic individuals and early diagnosis of patients with symptoms, which, in combination with multimodal treatment, has been shown to reduce breast cancer mortality by over 50% (10). Countries that have achieved sustained mortality reduction have done so through consistent breast cancer downstaging (diagnosis at Stage I or II) each year. Despite recent prioritization by Armenia’s Ministry of Health (MOH), Armenia has no organized screening strategy, and current screening practices remain limited in both reach and uptake (4, 6, 11). Longitudinal data on breast cancer staging, incidence, and mortality has not been evaluated, making it difficult to determine whether Armenia has been able to achieve sustainable downstaging and reductions in breast cancer mortality. Limited data suggests that 65-77% of cases are diagnosed in Stage I/II, and 2% are diagnosed as Ductal Carcinoma *In Situ* (DCIS) (5, 6).

To date, no study has evaluated the availability of diagnostic and treatment modalities for breast cancer in Armenia. In the present study, we conducted a facility-level national gap analysis of breast cancer care in Armenia by administering a structured, validated survey across four high-capacity tertiary institutions. The goal of this study is to identify existing system-level strengths and gaps foundational to early detection, timely diagnosis, and comprehensive management of breast cancer in Armenia, as outlined by the WHO-GBCI.

## Methods

### Study context

The Republic of Armenia is a country of three million people located in the Southern Caucasus of Asia. Approximately 36% of the population resides in the capital city, Yerevan, with the remainder distributed across marzer (provinces).

Armenia has made significant economic progress in the last 30 years of post-Soviet independence, attaining a UMIC designation by the World Bank in 2019 (12). Despite this progress, the Armenian healthcare system continues to face considerable challenges with modernization. While physician and hospital bed density is relatively high in the country, the Armenian health system faces a growing burden of noncommunicable diseases, with an estimated one in five Armenians expected to die prematurely from noncommunicable causes (6, 13).

### Study design

This cross-sectional descriptive study employs a breast cancer survey adapted from the Tanzania and Sergipe Breast Health Care Assessments (14, 15). The survey was translated into Armenian by a professional in-country translator at Yerevan State Medical University and reviewed for cultural and health-system appropriateness by local collaborators and study authors. In collaboration with administrators at the National Center of Oncology, three tertiary hospitals and one breast care facility in Yerevan were selected for participation in this study based on overall facility capacity and infrastructure, as well as the volume of breast cancer patients who present for services. Selected facilities included the National Center of Oncology, Erebuni Hospital, Astghik Medical Center, and the Armenian American Wellness Center. These sites were determined to be representative of the highest-capacity sites for breast cancer treatment in the country.

### Survey implementation

Surveys were administered in July 2025. Each facility was approached via email for participation. After initial electronic review of the instrument, the study team members conducted in-person site visits to complete the survey with institutional administrators at three sites; the fourth site submitted responses by email. Items covered institutional characteristics, patient volume and stage distribution, diagnostic and treatment infrastructure, pathology services, systemic and radiation therapy availability, palliative care, and financial arrangements for diagnostic and treatment services.

### Data analysis

Survey responses were entered into REDCap (Research Electronic Data Capture) for secure storage and cleaning. Descriptive summary statistics were used to characterize capacity across sites. Tabular summaries were constructed in Microsoft Excel. The study was reviewed by the UMass Chan Medical School Institutional Review Board (IRB), UCLA David Geffen School of Medicine IRB, and Yerevan State University IRB and determined to be exempt from human subjects research review.

## Results

### Patient demographics

At three of the four participating sites, a majority of breast cancer patients presented symptomatically rather than through screening referral (**Table 1**). Three sites were able to provide institutional stage-at-diagnosis data; of these, only one (Site 4) reported a Stage I/II proportion above 60%. DCIS accounted for 2-4% of diagnoses at reporting sites.

**Table 1:** Patient demographics and institutional stage distribution.

Survey Item	Site 1	Site 2	Site 3	Site 4
Patients presenting with symptoms of breast cancer	>75–90%	<50%	>50–<75%	>75–90%
Stage at diagnosis				
DCIS (%)	4	—	2	2
Stage I/II (%)	53	—	43	65
Stage III (%)	25	—	29	20
Stage IV (%)	19	—	26	13

### Financial responsibility for diagnosis and treatment

Patients bear the principal financial responsibility for diagnostic and therapeutic care at most sites, either through out-of-pocket payments or private insurance coverage (**Table 2**). Payment for the pathology report was patient-financed at two of four sites. Immunohistochemistry for estrogen receptor status, HER-2/neu testing, and fine-needle aspiration diagnosis were patient-financed at all three sites providing these data. Three sites agreed that cost was sometimes a barrier to starting or completing treatment, and that patients were at least sometimes required to obtain prescription drugs from external sources.

**Table 2:** Financial responsibility for diagnostic and therapeutic services.

Survey Item	Site 1	Site 2	Site 3	Site 4
Who pays for diagnostic services				
Pathology report	Patient	Government / Public insurance	Government / Public insurance	Patient
Immunohistochemistry (ER status)	Patient	Patient	Patient	Not available
HER-2/neu testing	Patient	Patient	Patient	Not available
Fine-needle aspiration (FNA)	Patient	Patient	Patient	Not available
Barriers to treatment				
Patient pays for chemotherapy if uninsured	—	Yes	Sometimes	Yes
Patients must procure drugs from outside sources	—	Sometimes	Sometimes	Yes
Patients do not start treatment due to cost	—	Sometimes	Sometimes	Sometimes
Patients do not complete treatment due to cost	—	Sometimes	Sometimes	Sometimes

“—” indicates that the facility did not provide a response for the survey item.

### Diagnostic capacity

All four sites reported having functional MRI, ultrasound, and CT capabilities (**Table 3**). Three sites operate an MRI with a dedicated breast coil. All four sites utilized PACS for image storage and review. All sites performed image-guided biopsy, core needle histology, and surgical biopsy. BRCA 1/2 testing and bone scanning were available at only two sites. Sentinel lymph node techniques, either blue-dye biopsy or radiotracer biopsy, were available at all sites. Gene profiling was available at two of four sites. Notably, only a single PET scanner is currently available nationwide, and is owned by one of the participating institutions; it is located near the hospital at a separate radiotherapy site.

**Table 3:** Diagnostic capacity across four high-volume centers in Yerevan.

Survey Item	Site 1	Site 2	Site 3	Site 4
Reporting and data				
BI-RADS for mammography	Yes	Yes	Yes	Yes
BI-RADS for ultrasound	Yes	Yes	Yes	Yes
BI-RADS for MRI	Yes	Yes	Yes	Yes
Imaging machines				
Functional PET machine	PET Scanner available at 1 site; not specified to preserve anonymity			
Functional MRI machines (n)	3	2	2	1
Picture archiving and communication system (PACS)	Yes	Yes	Yes	Yes
Functional ultrasound machines with >7 MHz transducer (n)	11	2	8	10
Ultrasound used to guide biopsy/FNA	Yes	Yes	Yes	Yes
MRI with dedicated breast coil	Yes	Yes	No	Yes
Functional CT scanner for breast cancer staging	Yes	Yes	Yes	Yes
Diagnostic techniques				
Clinical breast exam (CBE)	Yes	Yes	Yes	Yes
Ultrasound-guided FNA of suspicious axillary nodes	Yes	Yes	Yes	Yes
Sentinel lymph node biopsy with blue dye	Yes	Yes	No	Yes
Sentinel lymph node biopsy using radiotracer	No	No	Yes	Yes
Image-guided breast sampling	Yes	Yes	Yes	Yes
Preoperative needle localization (mammography/ultrasound)	Yes	Yes	Yes	Yes
Diagnostic breast ultrasound	Yes	Yes	Yes	Yes
Diagnostic mammography	Yes	Yes	Yes	Yes
Mammogram double reading	Yes	Yes	Yes	Yes
Breast MRI	Yes	Yes	—	Yes
Specimen radiography	Yes	No	Yes	Yes
Bone scan	No	No	Yes	Yes
BRCA 1/2 testing	No	No	Yes	Yes
Core needle tissue biopsy (histology)	Yes	Yes	Yes	Yes
Surgical biopsy	Yes	Yes	Yes	Yes
Pathology report content				
Tumor size, node status, histologic type, grade	Yes	Yes	Yes	Yes
TNM stage	Yes	Yes	Yes	Yes
ER status (immunohistochemistry)	Yes	Yes	Yes	Yes
PR status (immunohistochemistry)	Yes	Yes	Yes	Yes
Margin status determination	Yes	Yes	Yes	Yes
DCIS determination	Yes	Yes	Yes	Yes
HER-2/neu expression or amplification	Yes	Yes	Yes	Yes
Cytokeratin IHC of sentinel nodes (micrometastases)	Yes	No	Yes	Yes
Pathology double reading	Yes	Yes	Yes	Yes
Gene profiling tests	Yes	No	No	Yes
Pathology report logistics				
Typical pathology turnaround time	<1 week	<1 month	<1 week	<1 week
Report sent to outside referring provider/institution	No	Yes	Yes	Yes
Breast provider at institution receives report	Yes	—	Yes	—

BI-RADS, Breast Imaging Reporting and Data System; FNA, fine-needle aspiration; MRI, magnetic resonance imaging; PACS, picture archiving and communication system; SLN, sentinel lymph node. “—” indicates that the facility did not provide a response for the survey item.

Pathology report content was uniformly comprehensive — all sites reported inclusion of tumor size, nodal status, histologic type, grade, TNM stage, margin status, estrogen receptor (ER) and progesterone receptor (PR) status, HER-2/neu, and DCIS determination (**Table 3**). Pathology double reading was performed at all sites. Turnaround time was under one week at three sites and under one month at the fourth. Pathology reports were transmitted to referring external providers at three of four sites and were received by breast providers at the originating institution where that information was available.

### Treatment capacity

All four sites provide surgical management for breast cancer, including total or modified radical mastectomy, breast-conserving surgery, and breast reconstruction (**Table 4**). Sentinel lymph node biopsy approaches varied, with two sites offering both dye- and radiotracer-based techniques. Radiation therapy is provided at only two of the four sites, with variations in availability. Of these, one reported a linear accelerator (LINAC) but no Cobalt-60 unit, while the other did not provide information on its radiation infrastructure. Systemic anti-neoplastic drug therapy is offered at three of four sites, including 12 out of 13 of the surveyed anti-neoplastic drugs at all three; however, methotrexate was only available at one site.

**Table 4:** Treatment capacity across four high-volume centers in Yerevan.

Survey Item	Site 1	Site 2	Site 3	Site 4
Surgery — early-stage cancer				
Modified radical mastectomy	Yes	Yes	Yes	—
Breast-conserving surgery	Yes	Yes	Yes	Yes
SLN biopsy with blue dye	Yes	Yes	No	Yes
SLN biopsy using radiotracer	Yes	No	Yes	Yes
Breast reconstruction	Yes	Yes	Yes	Yes
Surgery — locally advanced cancer				
Modified radical mastectomy	Yes	Yes	Yes	Yes
Breast-conserving surgery	Yes	Yes	Yes	Yes
SLN biopsy with blue dye	Yes	No	No	Yes
SLN biopsy using radiotracer	Yes	Yes	Yes	Yes
Breast reconstruction	Yes	Yes	Yes	Yes
Surgery — metastatic or recurrent cancer				
Total mastectomy for ipsilateral recurrence after BCT	Yes	Yes	Yes	Yes
Radiation therapy offered				
Radiation therapy service	No	No	Yes	Yes
Cobalt-60 unit available	—	—	No	—
Linear accelerator (LINAC) available	—	—	Yes	—
Whole-breast irradiation for BCT (Stage I)	—	—	Yes	Yes
Post-mastectomy irradiation (Stage II, high risk)	—	—	Yes	—
Post-mastectomy irradiation (locally advanced)	—	—	Yes	Yes
Palliative radiation therapy	—	—	No	Yes
Systemic antineoplastic drug availability				
Systemic drug therapy service offered	No	Yes	Yes	Yes
Doxorubicin	—	Yes	Yes	Yes
Cyclophosphamide	—	Yes	Yes	Yes
Paclitaxel	—	Yes	Yes	Yes
Docetaxel	—	Yes	Yes	Yes
Methotrexate	—	No	No	Yes
5-Fluorouracil	—	Yes	Yes	Yes
Trastuzumab	—	Yes	Yes	Yes
Carboplatin	—	Yes	Yes	Yes
Capecitabine	—	Yes	Yes	Yes
Vinorelbine	—	Yes	Yes	Yes
Gemcitabine	—	Yes	Yes	Yes
Tamoxifen	—	Yes	Yes	Yes
Aromatase inhibitor (anastrozole/letrozole/exemest ane)	—	Yes	Yes	Yes
Chemotherapy drug shortages	—	Never	Sometimes	—
Tamoxifen prescribed to all women	—	No	No	No
Drugs prepared in functioning containment hood	—	Yes	Sometimes	Yes

BCT, breast-conserving therapy; LINAC, linear accelerator; SLN, sentinel lymph node. “—” indicates that the service category was reported as unavailable at the facility.

### Palliative care

Only one site reported availability of palliative care. This site endorsed providing pain management with NSAIDs, opioids (morphine and non-morphine options), co-analgesics (e.g. steroids), physical/occupational therapy, locoregional and spinal analgesia, and surgery. The site does not provide fentanyl patches or radiotherapy. Answer was not provided regarding availability of opioid pumps.

The site reported availability of a pain therapy specialist and home-based care. They endorsed sometimes having barriers to opioid therapy for pain relief due to drug shortages, laws restricting prescription amounts, physician reluctance to prescribe, patient reluctance to take them, and difficulty obtaining opioids for home use. The site endorsed that sometimes, strict control of morphine results in the use of weaker analgesics for patients with severe pain, and that physicians do not receive adequate education about opioid usage in pain management.

## Discussion

This study provides the first comprehensive, facility-level assessment of early detection, diagnostic, and treatment capacity for breast cancer across Armenia’s four highest-volume hospitals and oncology centers. We find that surveyed tertiary facilities in Yerevan broadly possess the fundamental technological and personnel resources required for modern breast cancer care. However, despite the availability of comprehensive diagnostic imaging and treatment services at these centers, Armenia’s reported MIR is disproportionately high, signaling that significant gaps exist along the breast cancer care continuum.

### Early detection, timely diagnosis, and multi-modal treatment capacity

Among sites able to provide institutional stage-at-diagnosis data, only one site reported a Stage I/II proportion above 60%, the WHO-GBCI early-detection benchmark. DCIS accounted for 2-4% of diagnoses at reporting sites, which is consistent with previous findings in Armenia, and substantially below the approximately 20% observed in nations with organized screening programs (16). Additionally, three sites reported that a majority of their patients presented with symptoms of breast cancer. These findings indicate that breast cancer cases in the surveyed facilities are predominantly detected after symptom onset rather than through preclinical screening. Without the implementation of organized, population-based screening, Armenia will face substantial challenges in achieving sustainable downstaging and meeting the WHO-GBCI benchmark for early-detection.

All sites reported functional mammography, ultrasound, MRI, and CT; three operated MRI with a dedicated breast coil; BI-RADS and PACS were uniformly used; and pathology reports uniformly included tumor size, nodal status, histologic type, grade, TNM stage, ER, PR, HER-2/neu, margin status, and DCIS determination. These findings compare favorably with regional and international surveys of diagnostic imaging capacity in post-Soviet and LMIC settings (17). Pathology reports are completed within a month for the majority of patients at all sites, falling within the 60 day WHO-GBCI benchmark for timely diagnosis in imaging and pathology. The delays in early detection, as noted above, are therefore more likely distributed across upstream patient facing processes (early symptom recognition, primary care screening/referral, and navigating multiple institutions to complete workup) (6).

Surgical capacity was the most uniformly robust component of the care continuum. All four sites offered modified radical mastectomy, breast-conserving surgery, and breast reconstruction, and all provided operative options for metastatic or recurrent disease. Surgery remains the backbone of curative-intent breast cancer management, and its broad availability across Armenia’s largest centers should, in principle, support timely operative management for the majority of patients presenting to these institutions.

Radiation therapy, in contrast, was available at only two of four sites. Of those, only one reported a LINAC and no Cobalt-60 unit, and the other did not provide radiation infrastructure information. Breast-conserving therapy requires postoperative whole-breast irradiation to maintain oncologic equivalence with mastectomy, and locally advanced disease commonly requires postmastectomy irradiation of the chest wall and regional nodes. This results in a potential bottleneck effect in curative-intent care delivery, which may contribute both to treatment delays and to decisions to opt for mastectomy when breast-conserving therapy would otherwise be appropriate. Prior regional assessments have similarly identified radiation oncology as one of the most constrained components of cancer care in the South Caucasus and central Asia (17).

For systemic therapy, twelve of thirteen surveyed antineoplastic agents were available at all three sites offering chemotherapy, with the exception of methotrexate, which was available at one site. The absence of methotrexate at the other two sites likely reflects intentional deprioritization of CMF-based regimens rather than a procurement failure, given the comprehensive availability of the other antineoplastic agents across all three sites, which cover all preferred and most other recommended regimens in contemporary breast cancer management. It is important to note, however, that institutional formulary capacity does not necessarily equate to patient-level access — drugs must be formally registered with the government and renewed periodically. A recent study showed that roughly 30% of drugs on Armenia’s national essential medicines list were not registered, making them unavailable to patients (6).

In the present study, we did not assess variables relevant to the third WHO-GBCI benchmark, which aims to achieve treatment completion and successful return home for 80% of patients. Overall, Armenia’s MIR implies a high rate of unsuccessful achievement of remission in the country. It is likely that some patients are lost to follow-up and do not complete treatment due to various barriers to healthcare access, chief among them being financial cost and a fractured care pathway which places onus on patients to seek diagnostic and treatment modalities across multiple facilities (6). Additional investigation is necessary to assess the extent of the impact attributable to these factors.

Overall, these results demonstrate the availability of comprehensive multimodal breast cancer treatment at the highest-volume tertiary centers in Armenia. However, the availability of services does not account for utilization, quality of care, or patient outcomes. Additionally, while the present study provides evidence related to the WHO-GBCI benchmarks for countries, data is insufficient to make claims about whether Armenia is or is not meeting the outlined criteria.

### Palliative care capacity

Palliative care was unavailable at 3 sites, and the fourth cited multiple barriers to opioid pain management for palliative patients, including lack of formal training and dedicated resources. This pattern is consistent with global trends in lower income countries, where palliative care is often the last component of the cancer-care continuum to be formally institutionalized. Given Armenia’s reported MIR, palliative care capacity should be expanded at high-volume centers for those patients who require end-of-life care.

### Barriers to care

One of the most consistent findings across sites was the financial burden on patients, even at the country’s highest-capacity centers. At three of four sites, patients paid out-of-pocket for immunohistochemistry and HER-2/neu testing, which are prerequisites for systemic therapy decision-making and therefore not elective. Pathology reporting was patient-financed at two sites. Three sites indicated that cost was at least sometimes a barrier to starting or completing chemotherapy, and that patients were at least sometimes required to procure drugs from external sources. These patterns are consistent with prior national estimates of high out-of-pocket health expenditure, and with modeled estimates suggesting that non-metastatic breast cancer care alone would consume approximately 1.6% of Armenia’s national health budget if fully publicly financed (7, 18). Moreover, state coverage for chemotherapy is capped at approximately $750 per year for vulnerable patients and half that for others, leaving most patients responsible for a substantial out-of-pocket remainder (6). Trastuzumab became available free of charge in 2020 for women with non-metastatic HER2/neu-positive breast cancer, but this remains an isolated provision rather than part of a broader coverage framework.

Armenia’s transition to universal health coverage (UHC), initiated in 2026, has the potential to shift these dynamics substantially (8). However, the effect will depend critically on the inclusion and depth of coverage for the diagnostic, molecular, and systemic therapy elements of breast cancer care. If reforms emphasize inpatient care and surgical episodes, but underfund outpatient diagnostics and oral systemic agents (which are the items most frequently patient-financed in our data), the pattern of patient-financed essential care may persist. Ongoing surveillance of stage distribution and treatment completion will be essential to evaluate the population-level impact of UHC implementation.

Geographic access is a parallel concern. Women who reside outside of Yerevan, which is home to all four participating sites surveyed in the present study, presumably face additional costs for periodic transportation to the city and time taken off of work to pursue diagnosis and treatment. These women may therefore be at an increased risk of delayed diagnosis and loss to follow-up.

### Limitations

This study was intentionally designed to survey Armenia’s highest-volume, highest-capacity cancer care centers in order to characterize maximum system-level capacity for breast cancer care, rather than provide a nationally representative estimate. As a result, the findings of this study do not capture resource constraints present in secondary hospitals, regional facilities, or rural primary care settings where initial screening and diagnostic decisions commonly occur. Sites were selected based on recommendations made by local collaborators at Armenia’s National Center of Oncology, and thus subject to selection bias. Moreover, the survey instrument does not assess real-world utilization, timeliness, completion of diagnostic and treatment modalities, quality of care, or patient-level outcomes. As a result, inferences regarding care delivery in practice should be made cautiously.

Institutional data were self-reported by key informants and subject to recall bias. Quantitative questions (**Table 1**) were answered utilizing the most recent data available at the time of survey implementation, and cannot be used to accurately predict longitudinal patterns. Some survey items were not answered by participating sites, which limits cross-site comparisons in specific domains. The survey did not directly quantify patient attrition or loss to follow-up, so inferences regarding care continuity are based on system-level characteristics rather than measured patient outcomes.

Subsequent phases of this project, currently underway, are designed to address these gaps by characterizing care delivery across a broader range of settings, including facilities where access barriers and resource limitations are likely more pronounced. Future studies will better evaluate gaps in screening or referral, which likely contributes to delayed detection and therefore delayed care, and will assess the extent of loss to follow-up for breast cancer patients navigating their care. Finally, a longitudinal assessment of national breast cancer data in Armenia is required to better assess trends in breast cancer incidence, mortality, and staging.

### Conclusion

Armenia’s highest-capacity cancer care centers possess the imaging, pathology, and genetic testing necessary to efficiently diagnose and characterize breast cancer. They additionally possess comprehensive treatment modalities including surgery, systemic drug therapy, and, at two sites, radiation. Despite the availability of these modern modalities in surveyed centers in Armenia, pathways for screening, referral, diagnosis, and treatment are fragmented, placing the onus on patients to follow-up care at multiple facilities. Patient out-of-pocket spending on essential diagnostic and therapeutic services, the absence of a national cancer registry, uneven radiation therapy distribution, and underdeveloped palliative care integration emerge as the principal system-level areas for improvement.

Addressing these gaps will require policy reform that prioritizes breast cancer downstaging through early detection. This includes establishing organized, population-based breast cancer screening; standardizing referral and follow-up pathways that close the loop between primary care, tertiary centers, and pathology services; creating a functioning national cancer registry to enable ongoing surveillance and policy evaluation; and ensuring financial protection mechanisms, to reduce the cost of care for patients. These system-level interventions are necessary to reduce breast cancer mortality in Armenia.

## Data Availability

The raw data supporting the conclusions of this article will be made available by the authors, without undue reservation.
